# Caring for Older Adult Parents: An Urban Malaysian Perspective

**DOI:** 10.1093/geroni/igaf122.4085

**Published:** 2025-12-31

**Authors:** Karuna Thomas

**Affiliations:** Washington University in St. Louis, St. Louis, Missouri, United States

## Abstract

This study set out to understand the experience of family caregivers in Malaysia who chose to coreside with their older adult parent in order to provide care. With an increasing aging population in Malaysia, along with the lack of policy supporting caregivers, this raised the question of how family caregivers manage the multiple roles in adulthood, along with the responsibilities that come with caring, and cultural expectations of filial piety as discussed in the literature. Twelve qualitative interviews were conducted to explore this experience among urban Malaysians who were coresiding with their parent in order to provide care. Participants were aged between 42 and 59 years old, and answered questions about their decision to coreside and how they manage responsibilities. Thematic analysis revealed that family caregivers are influenced by an ingroup decision making perspective with care of older adults being considered an important part of their cultural identity. Malaysian caregivers expressed a blindness to the burden associated with caregiving in which the responsibilities were viewed as a blessing and not a burden. Caregivers adjust to managing lifestyle changes by employing action-focused, emotion-focused, and spiritual-focused coping strategies. These findings provide further insight into how Malaysian caregivers perceive their experience and how policy can best support caregivers in the country.

